# Protective effects of amoxicillin and probiotics on colon disorders in an experimental model of acute diverticulitis disease

**DOI:** 10.1007/s10787-022-01093-w

**Published:** 2022-11-01

**Authors:** Maha G. Soliman, Hanaa A. Mansour, Wedad A. Hassan, Eman Shawky

**Affiliations:** 1 Department of Zoology, Faculty of Science, Al-Azhar University, Cairo, Egypt; 2 Department of Pharmacology, National Organization for Drug Control and Research, Giza, Egypt

**Keywords:** Amoxicillin, Probiotics, DSS, LPS, Colon disorders, Acute diverticulitis

## Abstract

Acute diverticulitis disease is associated with inflammation and infection in the colon diverticula and may lead to severe morbidity. This study aimed to evaluate and compare the protective effects of amoxicillin antibiotic, either alone or in combination with probiotics (*Lactobacillus acidophilus* and *Bifidobacterium lactis*), in a rat model of acute diverticulitis disease. Acute diverticulitis was induced, in albino rats, by adding 3% weight/volume of dextran sulfate sodium (DSS) to the rats’ drinking water; daily for 7 days, in addition to injecting lipopolysaccharide (LPS) enema (4 mg/kg). The impact of treatments was assessed by measuring the physiological and immunological parameters and evaluating colon macroscopic and microscopic lesions. The results showed that both treatments (especially probiotics with amoxicillin) alleviated the adverse effects of DSS and LPS. This was obvious through the modulation of the rats’ body weight and the colon weight-to-length ratio. Also, there was a significant (*p* < 0.001) decrease in the colon macroscopic lesion score. The pro-inflammatory cytokines [(TNF)-α, (IL)-1β, (IFN)-γ, and (IL)-18]; in the colon tissue; were significantly (*p* < 0.001) decreased. Also, both treatments significantly ameliorated the elevation of myeloperoxidase activity and C-reactive protein levels, in addition to improving the histopathological alterations in the colon tissue. In conclusion, amoxicillin and probiotics–amoxicillin were effective in preventing the development of experimentally induced acute diverticulitis, through their anti-inflammatory and immunomodulatory effects. Furthermore, this study has explored the role of probiotics in preventing DSS/LPS-induced acute diverticulitis, so it can be applied as a promising treatment option for acute diverticulitis disease.

## Introduction

Acute diverticulitis is a gastrointestinal disease, which is characterized by inflammation of the colon diverticulum and its surrounding mucosa (Strate et al. 2015). Diverticulitis refers to the diverticular disease, as well as the spectrum of complications associated with colon diverticulosis, such as bleeding (Cohen et al*. *2013). The colon diverticulum is formed as a consequence of herniation of the colon mucosa and submucosa through the perivascular connective tissue sheath, which surrounds the intramural vasa recta (Wedel et al*.* 2015). Part of the pathogenesis returns to deterioration in the colon wall strength in aged individuals, which may be due to the increased collagen cross-linkage of the colon wall (Wess et al. 1996). Increasing age also can lead to internal anal sphincter (IAS) dysfunction, characterized by a decrease in IAS tone and contractility (Singh and Rattan 2021). Recent studies have demonstrated that brain-derived neurotrophic factor (BDNF) has neuromuscular effects on basal IAS smooth muscle tone, which in turn evidenced its role in the pathophysiology of ageing-associated rectoanal motility disorders (Singh et al. 2020, 2022). Yamamichi et al. (2015) demonstrated age, gender, tobacco usage, obesity, pre-diabetic conditions, alcohol intake, and increased serum triglyceride concentrations; as risk factors for diverticular disease.

Acute diverticulitis initiates to develop when a colon diverticulum becomes obstructed by faeces, resulting in faecal stasis (Strate and Morris 2019). The impacted faeces lead to an obstruction of the lumen, raising the diverticular pressure by continuing mucus formation, and ultimately ulceration within the diverticular mucosa. These events allow for a proliferation of bacteria, diverticulum distension, and localised ischaemia (Vermeulen et al. 2010). When bacterial contamination over-reaches the peritoneal cavity, it leads to intra-abdominal sepsis and peritonitis (Ceresoli et al. 2018).

The genetic factors, as well as alterations in the colonic neuromuscular system, take part in the development of diverticulitis (Strate and Morris 2019). Furthermore, it was hypothesized that diverticular disease results from the deficiency of dietary fibres. According to this theory, the low-fibre diet causes small-calibre stools to increase the pressure inside the colon, and ultimately, herniation of the colon mucosa and submucosa through the muscle layers of the colon wall, which are adjacent to the vasa recta (Painter and Burkitt 1971). Diverticular disease has been associated with the alterations of bacterial flora; in the peri-diverticula, which cause diverticular inflammation as well as abdominal symptoms (Lahner et al*.* 2016). The alterations of bacterial flora, which occur as a result of slow colonic transit and faecal stagnation, impair the mucosal barrier function and enhance the release of inflammatory cytokines, which ultimately lead to colonic inflammation (Tursi 2007). In response to bacterial invasion, the colon’s epithelial cells release inflammatory cytokines. The inflammatory cytokines, such as tumour necrosis factor-alpha (TNF)-α and interferon-gamma (IFN)-γ, have been shown to increase intestinal permeability (Corridoni et al. 2012).

Due to the concept that diverticulitis is inflammatory as well as an infection-associated disease (Strate and Morris 2019) and since bacteria are responsible for inflammation, antibiotics have been considered the cornerstone for the treatment of acute diverticulitis. Moreover, because the colon harbours so many bacterial species, broad-spectrum antibiotics are prescribed treatment that targets a broad range of bacteria, including *Bacteroides* as well as other anaerobic bacteria. (Feingold et al*.* 2014). Amoxicillin is a bactericidal β-lactam antibiotic that is most widely used in Europe. Amoxicillin affects both Gram-positive and Gram-negative bacteria by inhibiting an enzyme essential for bacterial cell wall synthesis (Graversen et al. 2020). Inpatient treatment of acute diverticulitis encompasses intravenous fluid resuscitation, intravenous antibiotics, and bowel rest (Daniels et al. 2014). The outpatient treatment of mild or uncomplicated acute diverticulitis follows new guidelines, for the management of acute diverticulitis, which suggests that; antibiotics can be used selectively rather than routinely (Balasubramanian et al. 2017). Treatment of acute diverticulitis should target the acute inflammation in the colon diverticula. According to the extent of inflammation and over-reaching the peritoneal cavity, several approaches have been proposed. Antibiotic therapy is always mandatory due to the presence of bacterial contamination through the colon diverticula (Ceresoli et al. 2018). Probiotics have been reviewed, by different authors, in the management of diverticular disease (Ojetti et al. 2018). The authors have demonstrated that probiotics exert beneficial effects in the prevention and treatment of several gastrointestinal diseases through modulation of the gut microbiota composition (Rondanelli et al. 2017). *Lactobacillus* and *Bifidobacterium* are two types of probiotics that are extensively observed in the human intestine. The previous studies demonstrated that; these probiotic strains have anti-inflammatory and immunomodulatory activities (Yao et al. 2017). Furthermore, it was observed that; *Bifidobacterium adolescentis*, *Lactobacillus*, and *Phascolarctobacterium* were reduced in patients with intestinal inflammation. Interestingly, when they were present, they reduced inflammation by acting on C-reactive protein (CRP), interleukin (IL)-6, and (TNF)-α (Al Bander et al. 2020). In this study, data from a diverticulitis rat model highlight the critical involvement of dysregulated immune responses and impaired colonic epithelial defence system in the pathogenesis of acute diverticulitis disease. In addition, this study aimed to investigate the preventive and potential therapeutic value of antibiotics and probiotics in a rat model of acute diverticulitis disease.

## Materials and methods

### Chemicals and drugs

Dextran sulfate sodium (DSS) salt, (molecular weight: 40,000), was purchased from (Alfa Aesar, ThermoFisher, Kandel, GmbH, Germany); Lipopolysaccharide (LPS) was purchased from (Sigma-Aldrich, St. Louis, MO, USA); Probiotics (*Lactobacillus acidophilus* and *Bifidobacterium lactis*) were purchased from (PharmaCare Europe Ltd, West Sussex, RH10 9NQ, UK). Amoxicillin antibiotic was purchased from (Egyptian International Pharmaceutical Industries Company, Industrial Area, Egypt). DSS and LPS were dissolved in distilled water. Amoxicillin was suspended in distilled water, and shaken well before treatment. The probiotics pellets were dilacerated using a ceramic mortar and pestle; then, suspended in distilled water, and shaken well before treatment.

### Experimental animals

Male albino rats (Sprague Dawley), weighing 150–160 g, obtained from the animal house of the (National Organization for Drug Control and Research, Giza, Egypt); were used in the present study. The rats were housed in the laboratory room for one week before the commencement of the experiment; under controlled environmental conditions; constant temperature (25 ± 2 °C), humidity (60 ± 10%), and alternating 12 h of light/dark cycles. Standard pellet diet and water were allowed ad libitum. All animal procedures were performed following the Institutional Ethics Committee and under the recommendations for the proper care and use of laboratory animals. The study was approved by the Ethics Committee for Animal Experimentation of Cairo University with approval number (CUIF6219). Unnecessary disturbance of animals was avoided. Animals were treated gently; squeezing, pressure, and tough manoeuvres were avoided.

### Experimental design

The rats were randomly divided into seven groups, with six rats in each group, for a study period of 7 days, as following: group I, control group: rats only received water and food (no treatment); group II, DSS group: rats were received 3% DSS solution, added to their drinking water, daily for 7 days; group III, LPS group: rats were injected with LPS enema, by a catheter, at a dose of (4 mg/kg), 48 h before sacrificing them at the end of the experiment; group IV, amoxicillin-treated group: rats were treated with amoxicillin (0.162 g suspended in 2 ml distilled water/rat, by oral gavage, once daily for 7 days; group V, DSS/LPS group: in which acute diverticulitis was induced, rats were received DSS and LPS enema, similar to the ways mentioned above, in DSS and LPS groups; group VI, DSS/LPS-amoxicillin-treated group: rats were received DSS, LPS, and amoxicillin; at the doses and the ways mentioned in DSS, LPS, and amoxicillin groups; group VII, DSS/LPS–probiotics–amoxicillin-treated group: rats were received DSS, LPS, amoxicillin (at the doses and the ways mentioned in DSS, LPS, and amoxicillin groups), and probiotics (*Lactobacillus acidophilus* and *Bifidobacterium lactis*) each of which (4 × 10^8^ CFU suspended in 2 ml distilled water) orally, once daily for 7 days. The working doses of amoxicillin and probiotics (0.162 g and 4 × 10^8^ CFU, respectively) were selected based on a preliminary experiment performed on the rats that were treated with DSS/LPS. These rats were subdivided into DSS/LPS–amoxicillin-treated groups (where three doses of amoxicillin [0.081 g, 0.162 g, and 0.224 g] were tested); and DSS/LPS–probiotics-treated groups (where three doses of probiotics [2 × 10^8^ CFU, 4 × 10^8^ CFU, and 8 × 10^8^ CFU] were tested). These groups were assessed for body weight loss, colon weight/length ratio, stool score, and colonic macroscopic damage evaluation. Our preliminary data denoted that the most reasonable results were obtained in DSS/LPS–amoxicillin-treated group at the dose level (0.162 g) and DSS/LPS–probiotics-treated group at the dose level (4 × 10^8^ CFU), so they were adopted for accomplishing the subsequent experiments; in our study to explore their protective role against DSS/LPS-induced acute diverticulitis.

### Induction of acute diverticulitis

Acute diverticulitis was experimentally induced by adding 3% weight/volume of DSS, dissolved in distilled water, to the rats’ drinking water; daily for 7 days (Masubuchi and Horie 2004). Furthermore, the rats were injected with LPS enema, at the dose of (4 mg/kg), by a Nelaton catheter 8 FG, 48 h before sacrificing the rats; at the end of the experiment, where local immune reaction by LPS seems to play an important role in the perpetuation of experimental diverticulitis (Hotta et al. 1986) and aggravating colon inflammation (Zhang et al. 2011).

### Assessment of body weight loss, colon weight/length ratio, and stool score

The alteration in rats’ body weights was monitored from the beginning to the end of the experiment. The colons were excised, opened longitudinally, and washed with phosphate-buffered saline. The length of the colon was measured from the ileocecal junction to the anal verge. The colon weight-to-length ratio was calculated by dividing the colon weight (mg) by the colon length (cm) for each rat. The stool scoring criteria were performed according to Yamada et al. (2014) as following: stool consistency (0 = normal; 2 = loose stools; 4 = watery diarrhea) and the occurrence of gross blood in the stool (0 = negative; 4 = positive).

### Colon macroscopic lesions evaluation

Colonic damage evaluation was performed according to Cuzzocrea et al. (2003) as following: (0 = no injury; 1 = localized hyperemia; 2 = ulcers; 3 = ulcers with inflammation; 4 = extending ulcers with inflammation (more than 1 cm along the length of the colon); and 5 = ulceration covering 2 cm, the score is increased 1 point for each additional centimetre of ulceration covering the length of the colon.

### Blood sampling and colon tissue preparation

At the completion of the days of the experiment, the rats were anaesthetized with 4% isofluorane and sacrificed to enable blood and tissue collection. The serum was separated from the rats’ blood by centrifugation of blood at 4000 R.P.M. for 10 min, divided into aliquots, and stored at − 70 °C until used for analysis. The colons were excised from the ileocecal junction to the anus and the colon damage was evaluated macroscopically for each rat. Part of the colon from each rat (unique for all rats, 8 cm away from the anus) was separated and fixed in 10% neutral-buffered formalin for use in the histopathological examination. The rest of the colon tissues were wrapped in aluminium foil and kept frozen at − 70 °C until used for analysis.

### Determination of myeloperoxidase (MPO) activity in the colon tissue

MPO activity was evaluated using a kinetic colourimetric technique described by Bradley et al. (1982). The colon tissue homogenates were subjected to three cycles of freezing and thawing (− 70 °C/37 °C). Then, the homogenates were centrifuged at 10,000 rpm at 4 °C for 15 min. 50 µl of the supernatant were separated for use in the MPO assay. MPO activity was evaluated by adding and incubating 50 µl of the supernatant for 5 min at 37 °C to 2.4 millilitres of 50 mM potassium phosphate buffer (K_2_HPO_4_), PH 6.0, containing 0.167 mg/ml of ortho-dianisidine dihydrochloride, and 4.0 µl of 30% hydrogen peroxide (H_2_O_2_). Ortho-dianisidine is oxidized by MPO in the presence of H_2_O_2_ and produces a yellowish-orange product that can be absorbed at 460 nm. One unit of MPO activity is defined as that required to degrade 1 μmol of H_2_O_2_ per minute at 25 °C. The results were expressed as (U/g of tissue).

### Determination of serum C‐reactive protein (CRP) level

Serum CRP was measured using a kit from (Spinreact, Girona, Spain). The test is based on the principle of latex agglutination.

### Measurement of pro-inflammatory cytokines in the colon tissue

The levels of the colonic (TNF)-α, (IL)-1β, (IFN)-γ, and (IL)-18 were measured using a colourimetric sandwich enzyme-linked immunosorbent assay (ELISA) kit (MyBiosource, Inc., San Deigo, USA), according to the manufacturer instructions.

### Colon microscopic lesions evaluation

Colon tissues, fixed in 10% neutral-buffered formalin, were washed and dehydrated in ascending grades of alcohol. Next, the specimens were cleared in xylene, embedded in paraffin, sectioned into 4 μM thickness, stained with hematoxylin and eosin (H & E), and examined under a light microscope. Histological scores were assigned according to Vowinkel et al. (2004) as following: severity of inflammation (0 = no inflammation; 1 = slight inflammation; 2 = moderate inflammation; 3 = severe inflammation), depth of injury (0 = no injury; 1 = mucosal damage; 2 = mucosal and submucosal damage; 3 = transmural injury), and crypt damage (0 = no crypt damage; 1 = basal one-third damaged; 2 = basal two-thirds damaged; 3 = only surface epithelium intact; 4 = entire crypt and epithelium lost, and congested dilated vessels.

### Statistical analysis

Statistical differences between groups were computed by one-way analysis of variance (ANOVA) followed by the Tukey–Kramer test for multiple comparisons. The results were analyzed using the GraphPad Prism program (GraphPad software 5, San Diego, CA, USA). *P* < 0.05 was considered statistically significant. Data were expressed as mean ± standard error of the mean (SEM).

## Results

### Effect of three different doses of amoxicillin and probiotics on DSS/LPS-induced acute diverticulitis

**Fig. 1:**
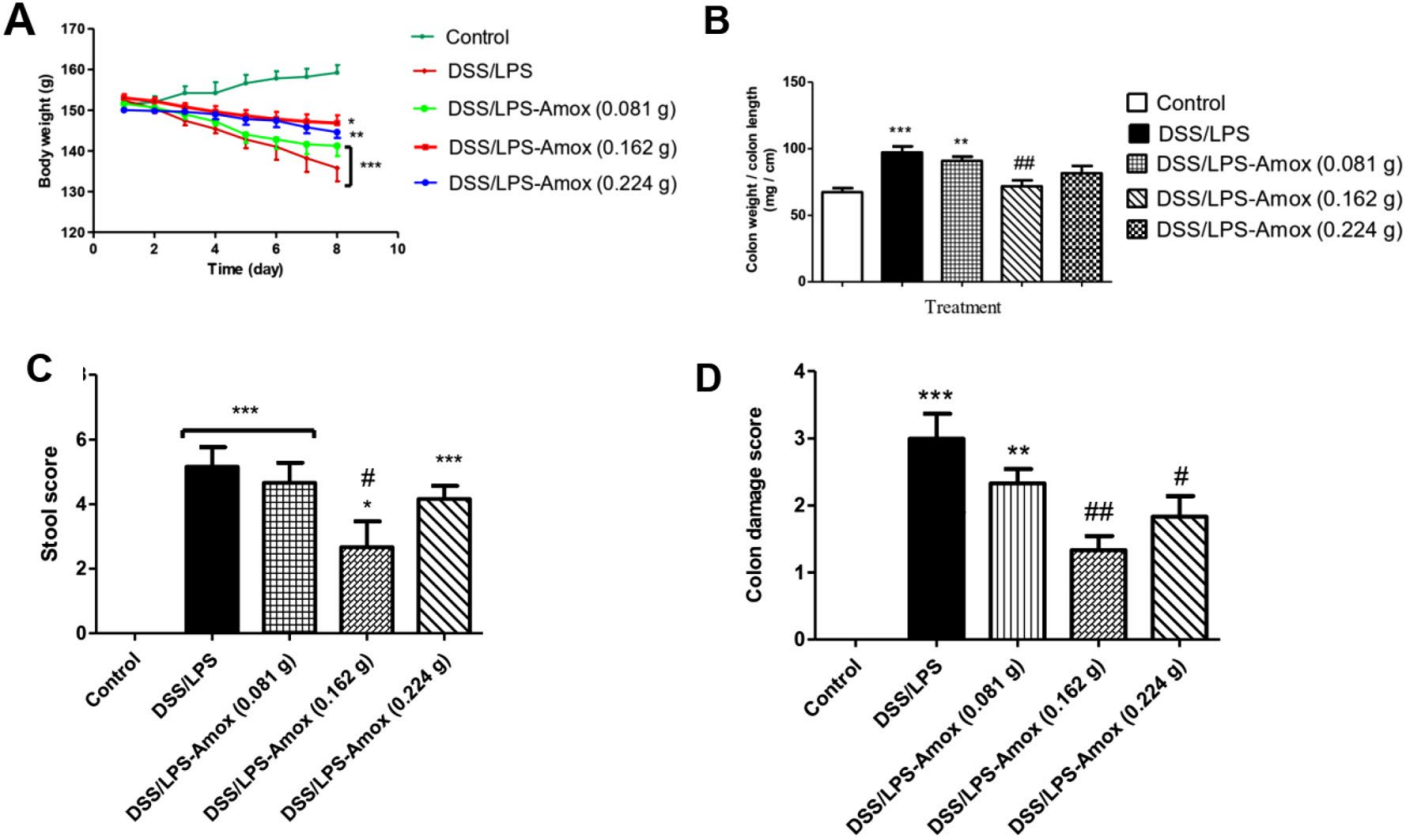
Effect of three different doses of amoxicillin on DSS/LPS-induced acute diverticulitis. **A** Rats’ body weight, **B** colon weight-to-length ratio, **C** stool score, and **D** colon macroscopic lesions score. Data are means, with their standard errors represented by vertical bars. Statistical analysis was performed using one-way ANOVA followed by the Tukey–Kramer test. *,** and *** represent significant differences from the control group (^*^p < 0·05, ^**^p < 0·01,^***^p < 0·001).^#, ##^ represent significant differences from DSS/LPS group (^#^*p* < 0·05, ^##^*p* < 0·01)

The preliminary experiment for the effect of three different doses of amoxicillin and probiotics on DSS/LPS-treated rats indicated that; amoxicillin at the dose level (0.162 g) induced a significant decrease (*p* < 0.01) in the colon weight-to-length ratio and the colon macroscopic damage score; and (*p* < 0.05) in the stool score; compared with DSS/LPS-treated rats. Amoxicillin at the dose level (0.224 g) only decreased the colon macroscopic damage score significantly (*p* < 0.05); compared with DSS/LPS-treated rats. Amoxicillin at the dose level (0.081 g) did not have any significant effect (*p* > 0.05) on rats’ body weight, colon weight-to-length ratio, stool score, and colon damage score; compared with DSS/LPS-treated rats (Fig. 1A–D).

**Fig. 2:**
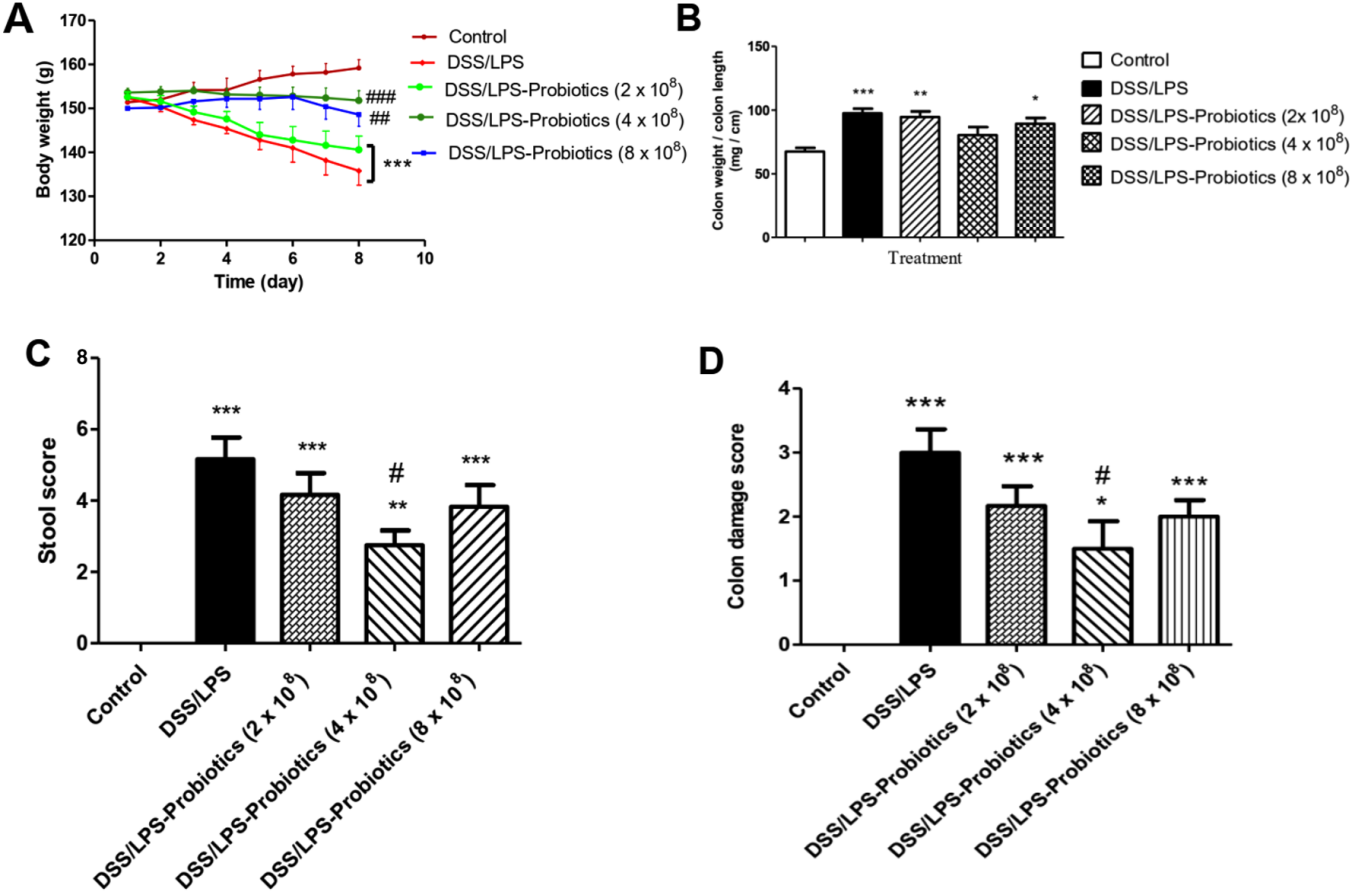
Effect of three different doses of probiotics on DSS/LPS-induced acute diverticulitis. **A** Rats’ body weight, **B** colon weight-to-length ratio, **C** stool score, and **D** colon macroscopic lesions score. Data are means, with their standard errors; represented by vertical bars. Statistical analysis was performed using one-way ANOVA followed by the Tukey–Kramer test. *,** and *** represent significant differences from the control group (^*^p < 0·05, ^**^p < 0·01,^***^p < 0·001).^#, ##^ and ^###^ represent significant differences from DSS/LPS group (^#^*p* < 0·05, ^##^*p* < 0·01, and ^###^*p* < 0·001)

Probiotics at the dose level (4 × 10^8^ CFU) resulted in a significant increase (*p* < 0.001) in the rats’ body weight; and a significant decrease (*p* < 0.05) in the stool score and colon damage score; compared with DSS/LPS-treated rats. Probiotics at the dose level (8 × 10^8^ CFU) only increased the rats’ body weight significantly (*p* < 0.01); compared with DSS/LPS-treated rats. Probiotics at the dose level (2 × 10^8^ CFU) could not induce any significant change (*p* > 0.05) in the rats’ body weight, colon weight-to-length ratio, stool score, and colon damage score compared with DSS/LPS-treated rats (Fig. 2A–D). According to these results, amoxicillin at the dose level (0.162 g) and probiotics at the dose level (4 × 10^8^ CFU) are the most appropriate doses.

### Effect of amoxicillin and probiotics–amoxicillin on rats’ body weight, colon weight/length ratio, and stool score

**Fig. 3:**
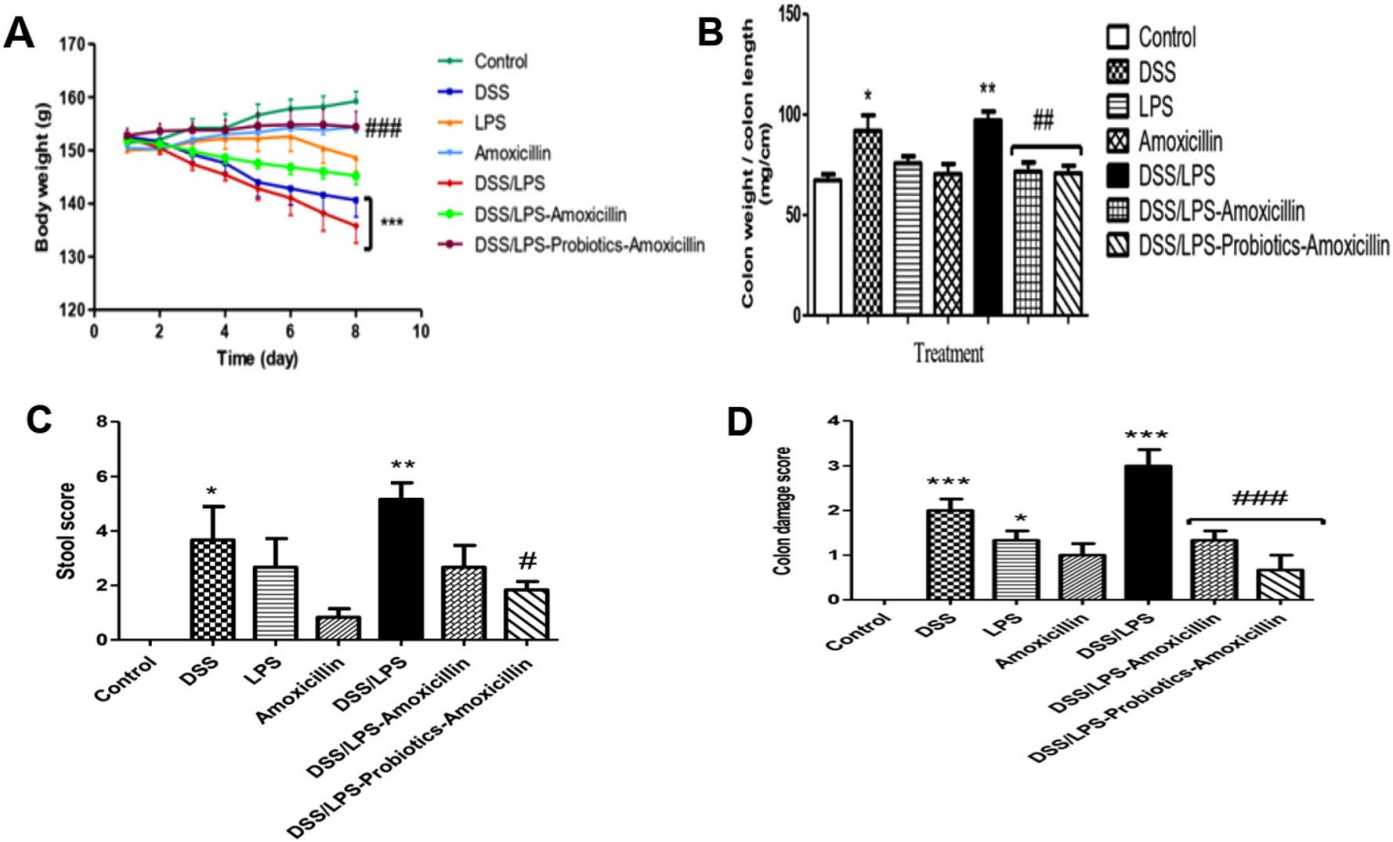
Effect of amoxicillin and probiotics–amoxicillin on DSS/LPS-induced acute diverticulitis. **A** Rats’ body weight, **B** colon weight-to-length ratio, **C** stool score, and **D** colon macroscopic lesions score. Data are means, with their standard errors; represented by vertical bars. Statistical analysis was performed using one-way ANOVA followed by the Tukey–Kramer test. *,** and *** represent significant differences from the control group (^*^p < 0·05, ^**^p < 0·01,^***^p < 0·001). ^**#**^,^**##**^ and ^**###**^ represent significant differences from DSS/LPS group (^**#**^*p* < 0·05, ^**##**^*p* < 0·01, and ^**###**^*p* < 0·001)

As shown in (Fig. 3A–C), the significance of the rats’ body weight loss (*p* < 0.001), a high colon weight-to-length ratio (*p* < 0.01), and a high stool score (*p* < 0.01); was demonstrated in DSS/LPS group, compared to the control group. Otherwise, treatment with amoxicillin, in DSS/LPS-amoxicillin-treated group, showed a significant decrease in the colon weight-to-length ratio (*p* < 0.01), a marked decrease in the stool score, while did not show a significant difference (*p* > 0.05) in the rats’ body weight loss; compared with DSS/LPS group. Furthermore, treatment with probiotics–amoxicillin significantly increased the rats’ body weight (*p* < 0.001), decreased the colon weight-to-length ratio (*p* < 0.01), and decreased the stool score (*p* < 0.05), compared with DSS/LPS group.

### Effect of amoxicillin and probiotics–amoxicillin on macroscopic lesions of colon

The colon tissues of rats; in the control group; exhibited a normal appearance, while those in the amoxicillin group displayed localized hyperemia. Localized hyperemia and ulcers were observed in the rats’ colons of DSS and LPS groups. In addition, localized hyperemia and extending ulcers with inflammation were observed in the rats’ colons of DSS/LPS group. Treatment of DSS/LPS-treated rats with amoxicillin showed inhibition of inflammation; however, there were localized hyperemia and ulcers. Moreover, probiotics–amoxicillin treatment, in DSS/LPS–probiotics–amoxicillin group, markedly reduced the colon macroscopic damage, since the colons did not exhibit any ulcers or inflammation, and there was only minimal hyperemia (Fig. 3D).

### Effect of amoxicillin and probiotics–amoxicillin on MPO activity and CRP level

**Fig. 4:**
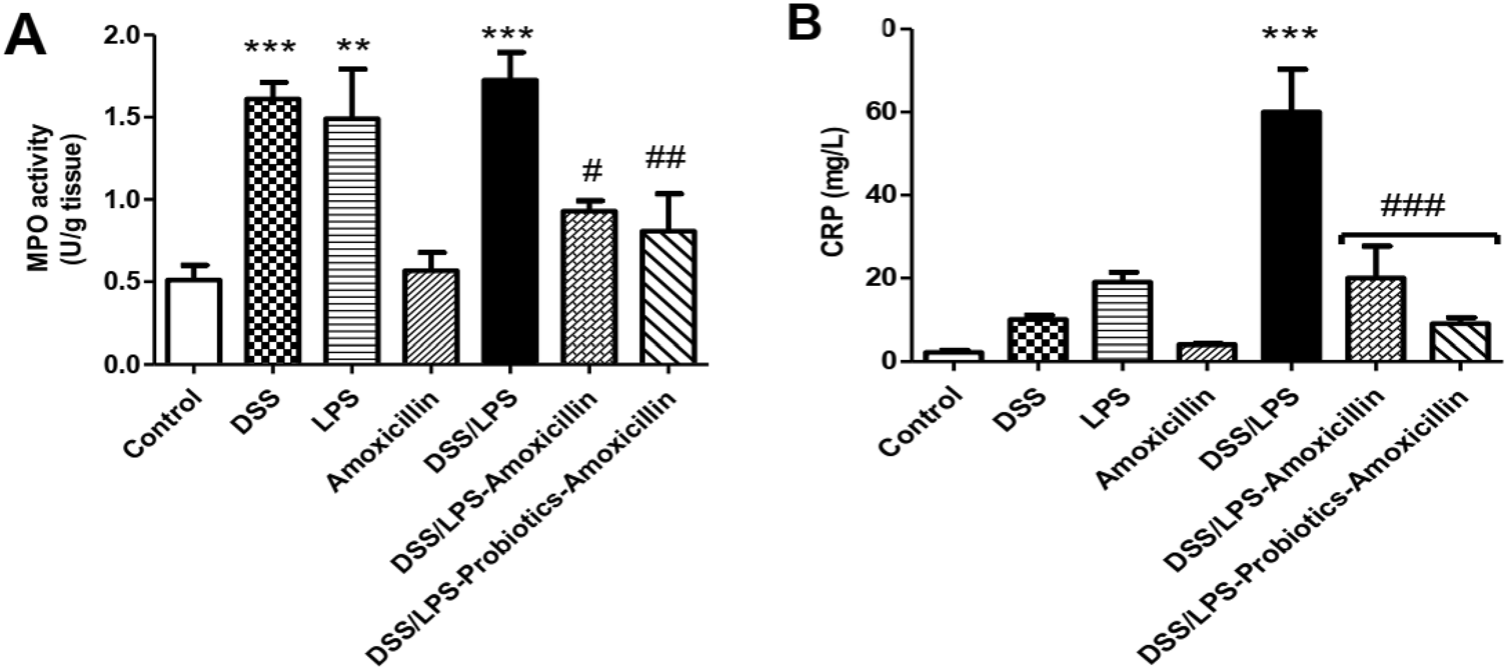
Effect of amoxicillin and probiotics-amoxicillin on DSS/LPS-induced acute diverticulitis. **A** MPO (myeloperoxidase) activity, in the colon tissue, **B** serum CRP (C-reactive protein) concentration. Data are means, with their standard errors; represented by vertical bars. Statistical analysis was performed using one-way ANOVA followed by the Tukey–Kramer test. ** and *** represent significant differences from the control group (^**^p < 0·01,^***^p < 0·001). ^**#, ##**^**,** and ^**###**^ represent significant differences from DSS/LPS group (^**#**^*p* < 0·05, ^**##**^*p* < 0·01, and ^**###**^*p* < 0·001)

Compared with the control group, MPO activity, in the colon tissue, was significantly elevated (*p* < 0.001) in DSS and DSS/LPS groups and (*p* < 0.01) in LPS group. On the other hand, MPO activity was significantly decreased (*p* < 0.05) by amoxicillin; and (*p* < 0.01) by probiotics–amoxicillin, compared to DSS/LPS group (Fig. 4A). Administration of DSS or LPS enema showed no significant difference (*p* > 0.05) in the serum CRP level; compared with the control group. Contrariwise, the serum CRP level was significantly increased (*p* < 0.001) by DSS/LPS; compared with the control group. Amoxicillin, with or without probiotics, reversed this increase in the serum CRP level; in comparison to DSS/LPS group (Fig. 4B).

### Effect of amoxicillin and probiotics–amoxicillin on pro-inflammatory cytokines in the colon tissue

**Fig. 5:**
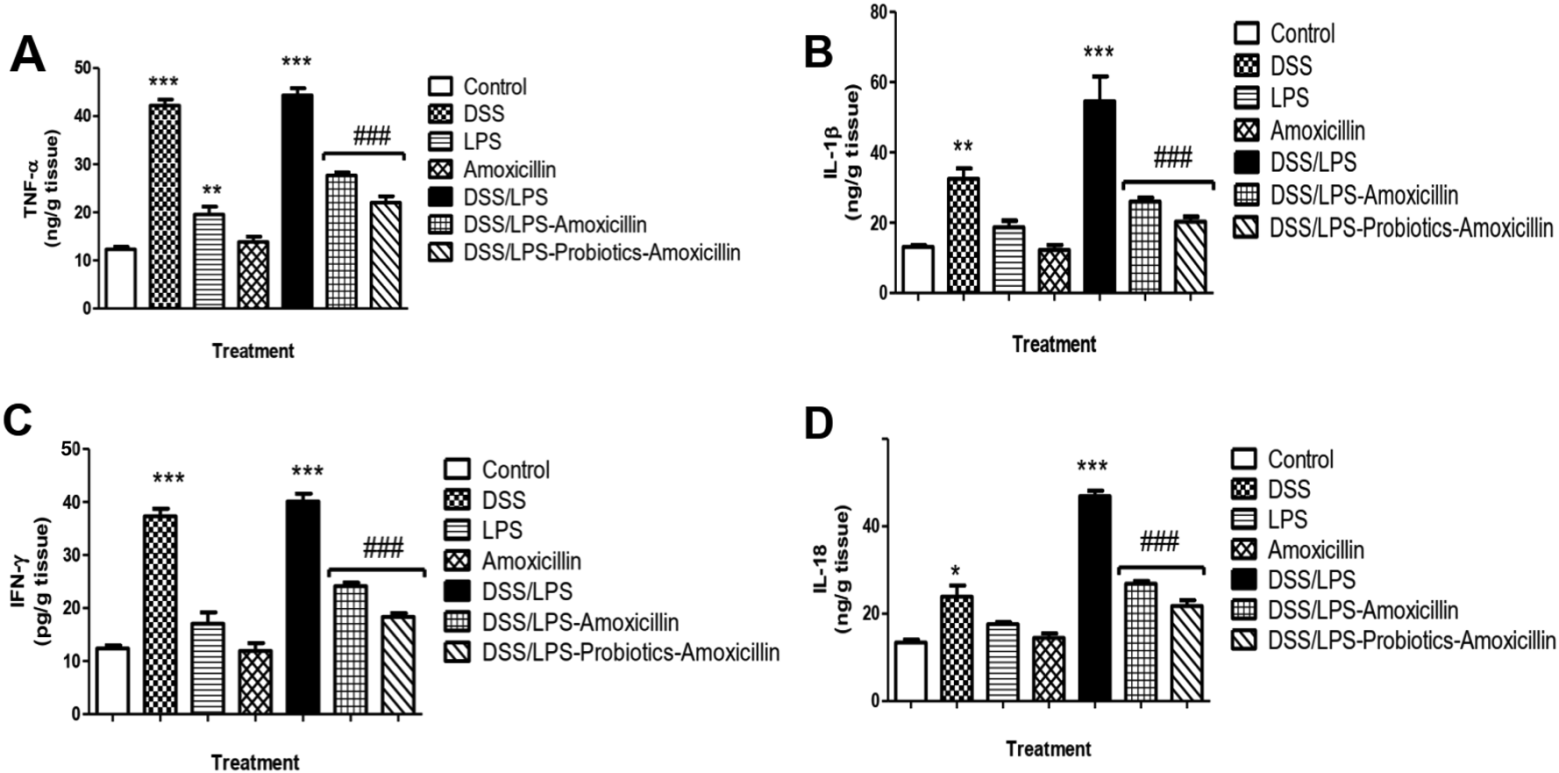
Effect of amoxicillin and probiotics-amoxicillin on DSS/LPS-induced acute diverticulitis. Levels of the pro-inflammatory cytokines **A** TNF-α (tumour necrosis factor-alpha), **B** IL-1β (interleukin-1 beta), **C** IFN-γ (interferon-gamma), and **D** IL-18 (interleukin-18), in the colon tissue, were determined by ELISA. Data are means, with their standard errors; represented by vertical bars. Statistical analysis was performed using one-way ANOVA followed by the Tukey–Kramer test. *,** and *** represent significant differences from the control group (^*^p < 0·05,^**^p < 0·01,^***^p < 0·001). ^**###**^ represents significant difference from DSS/LPS group (^**###**^*p* < 0·001)

The levels of the pro-inflammatory markers ](TNF)-α, (IL)-1β, (IFN)-γ, and (IL)-18)[ were significantly (*p* < 0.001) increased by DSS and DSS/LPS, compared with the control group. LPS enema only resulted in raising (TNF)-α level (*p* < 0.01); compared with the control group. Treatment with amoxicillin and probiotics–amoxicillin significantly (*p* < 0.001) ameliorated this increase; compared with DSS/LPS group (Fig. 5A–D).

### Effect of amoxicillin and probiotics–amoxicillin on colon histopathology

**Fig. 6:**
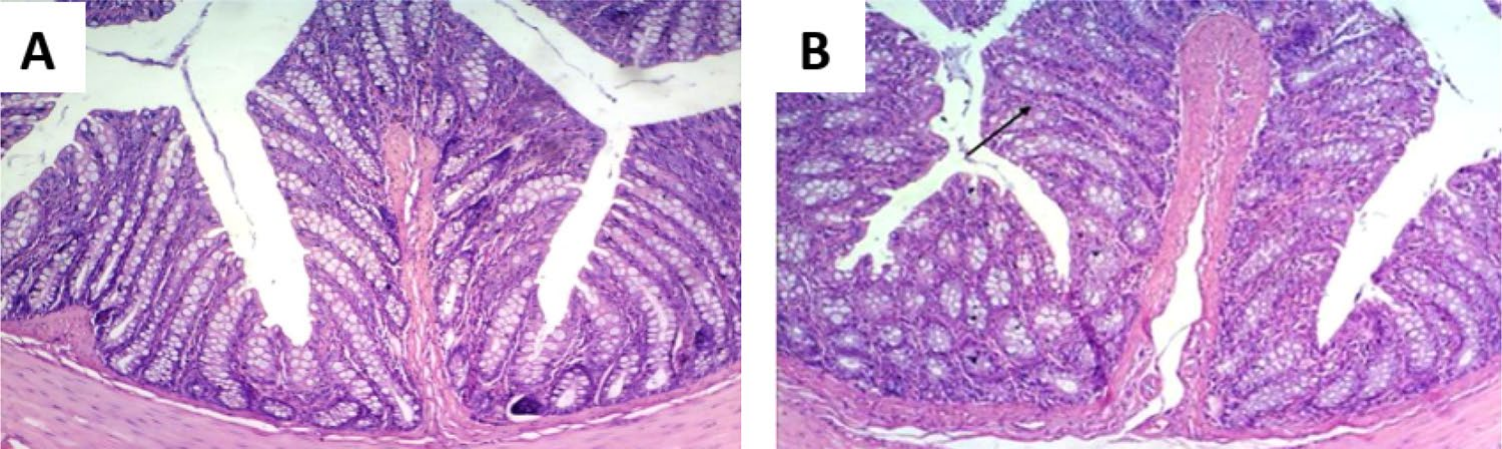
Photomicrograph of rat colonic tissue from **A** control group showing no histopathological alterations, **B** amoxicillin-treated group showing almost normal histopathological appearance with few cellular infiltrations (black arrow) (H & E 100×)

**Fig. 7:**
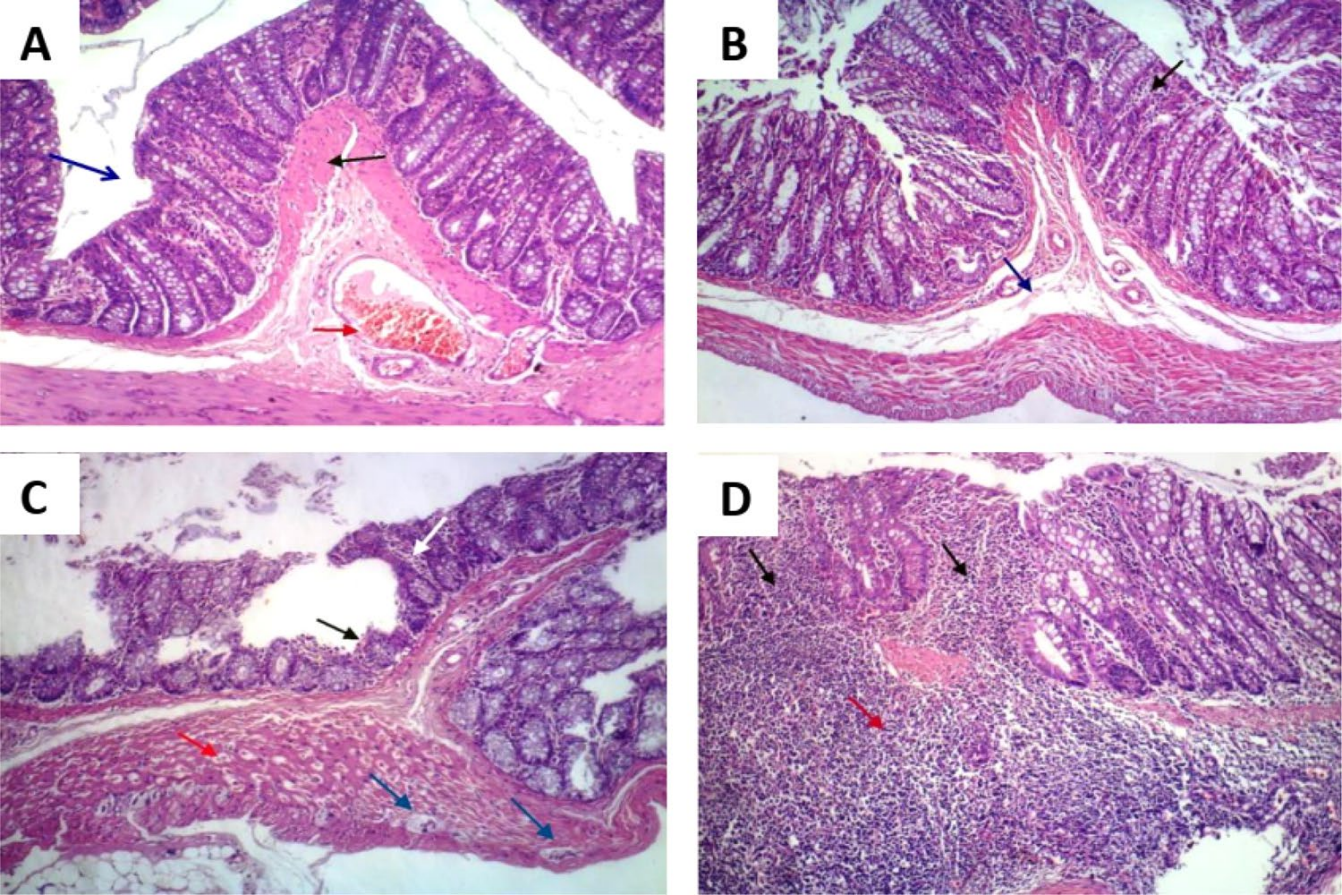
Photomicrograph of rat colonic tissue from **A** DSS group showing degenerative mucosal changes (blue arrow), hypertrophy of muscularis mucosa (black arrow), and congestion of submucosal blood vessel (red arrow), **B** LPS group showing few mucosal inflammatory cells infiltration (black arrow) and slight submucosal oedema (blue arrow), **C** DSS/LPS group showing mucosal degenerative changes (black arrow) with; damaged crypt, shortening of the mucosa, depletion of mucous glands, and inflammatory cells infiltration (white arrow); degeneration of the muscularis externa (red arrow), and formation of crypt abscesses (blue arrows), **D** DSS/LPS group showing degenerative mucosal changes with massive lymphocytes infiltration (black arrows) which extended to the submucosa (red arrow), (H & E 100×)

**Fig. 8:**
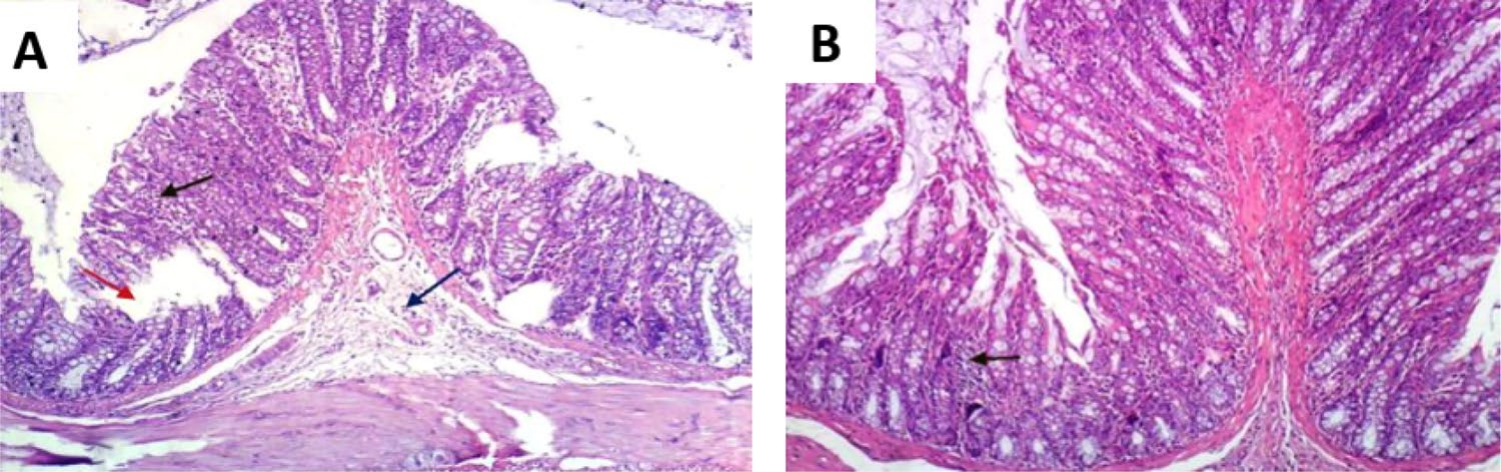
Photomicrograph of rat colonic tissue from **A** DSS/LPS–amoxicillin-treated group showing few mucosal (black arrow) and submucosal (blue arrow) inflammatory cells infiltration, and degenerative mucosal changes with damaged crypt (red arrow), **B** DSS/LPS–probiotics–amoxicillin-treated group showing few mucosal lymphocytes infiltration (black arrow) (H & E 100×)

**Fig. 9:**
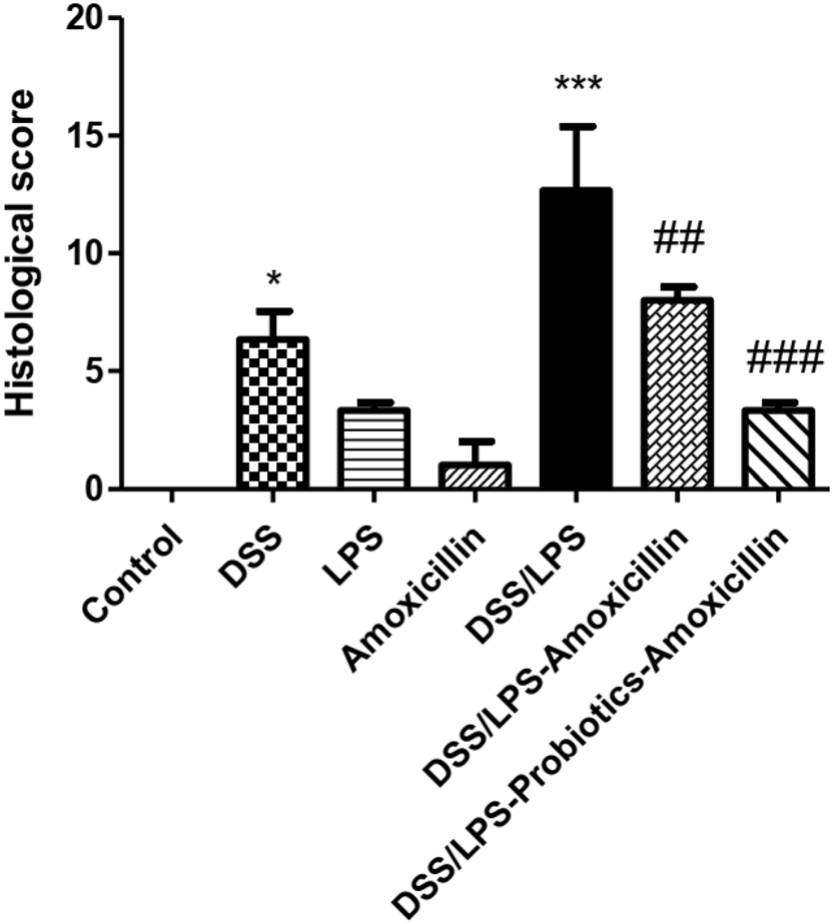
Total histological scores based on the histological grading criteria. Data are means, with their standard errors. Statistical analysis was performed using one-way ANOVA followed by the Tukey–Kramer test. *, and *** represent significant differences from the control group (^*^p < 0·05, ^***^p < 0·001). ^##, ###^ represent significant differences from DSS/LPS group (^##^*p* < 0·01, ^###^*p* < 0·001)

The colon tissues, stained with H and E, were clearly visible under the light microscope; and the different colon layers (mucosa, submucosa, and muscularis externa) were distinctly identified. The colon tissues of the control and amoxicillin groups did not show any observable histopathological changes as shown in (Fig. 6A, B). Contrariwise, colon tissues of DSS group showed moderate inflammation, mucosal and submucosal injury, and only surface intact (Fig. 7A). The colon tissues of LPS group displayed slight inflammation, mucosal damage, and basal crypt damage (Fig. 7B). Furthermore, the colon tissues of DSS/LPS group showed severe inflammation and lymphocytes infiltration, entire crypt and epithelium loss, and also there were congested dilated vessels (Fig. 7C, D). Conversely, treatment with amoxicillin revealed mucosal and submucosal inflammatory cells infiltration, dilated blood vessels, and degenerative mucosal changes with damaged crypts (Fig. 8A). The colon tissues of DSS/LPS–probiotics–amoxicillin-treated rats showed amelioration of the colon injury, slight inflammation, and few mucosal lymphocytes infiltration (Fig. 8B). The total histological scores were shown in Fig. 9 based on the histological grading criteria.

## Discussion

Diverticular disease refers to a spectrum of alterations in the intestinal tract, which begins with diverticulosis, herniation of mucosa and submucosa through the muscle layer of the colon wall, commonly in regions where the mural blood vessels penetrate through the muscle layer of the colon wall. (Kleessen et al. 1999). In terms of diverticulitis pathogenesis, it has been proposed that; diverticulitis develops from obstruction of a diverticulum’s neck, resulting in bacterial proliferation, local ischemia, and micro-perforation (Jacobs 2007). Thus, dysbiosis is considered to be an important determinant in the pathogenesis of diverticulitis; since the imbalance in the microbial milieu leads to disruption of the immune homeostasis and causes intestinal diseases (Chassaing and Darfeuille-Michaud 2011). Otherwise, the interactions between the intestinal flora and the host immune system have a critical role in the prevention of intestinal diseases, where the commensal microbiome enhances the maturation of the mucosal immune system, whereas the pathogenic microbiome causes immunity dysfunction which leads to disease development (Shi et al*.* 2017). As a consequence, using antibiotics or probiotics to manipulate the gut microbiota composition has recently been recommended as a therapeutic strategy for acute diverticulitis disease. Antibiotics have long been the cornerstone for the management of acute diverticulitis, intending to prevent inflammation and alleviate-associated symptoms (Hanna and Kaiser 2021). Moreover, antibiotic therapy has been recommended as an immunotherapy strategy for patients with colon cancer (Li et al. 2022). Amoxicillin, the most widely used penicillin, binds to the penicillin-binding protein (PBP) 1A, which is essential for bacterial cell wall synthesis. Amoxicillin's β-lactam ring opens to acylate the transpeptidase C-terminal domain of PBP 1A. This irreversible binding inactivates PBP 1A, which is involved in the final stages of the synthesis of peptidoglycan, the major component of bacterial cell walls. Inhibition of PBPs leads to defects in the bacterial cell wall structure, leading ultimately to bacterial cell lysis and death (Huttner et al. 2019). In addition, clinical trials have confirmed that; probiotic strains could reduce the side effects of cancer-related microbiota dysbiosis. Probiotics also can be used to treat several health conditions, such as inflammation, diarrhoea, irritable bowel syndrome, infections, and cancers, since they produce folate and bacteriocin. Bacteriocin is a low molecular weight protein with anti-inflammatory, anticancer, and immunomodulatory properties (Sankarapandian et al. 2022).

In this study, we showed that amoxicillin (especially together with probiotics) had protective effects against DSS/LPS-induced acute diverticulitis. DSS induces intestinal inflammation, which damages the epithelial monolayer lining the large intestine, allowing the dissemination of pro-inflammatory intestinal contents (e.g. bacteria and their products) into the underlying tissue (Chassaing et al. 2014). Furthermore, LPS aggravates colon inflammation (Zhang et al. 2011). In this study, DSS together with LPS enema gave rise to a significant decrease in the rats’ body weight. This loss in body weight is due to a deficiency of nutrients resulting from food aversion, reduced appetite, malabsorption, as well as loss of body fluids through colorectal bleeding and diarrhoea (Hunschede et al. 2017). Also, there was an increase in the colon weight-to-length ratio due to tissue oedema, necrosis, and inflammatory cell infiltration confirmed in our study through the high macroscopic and microscopic lesion scores. These results are consistent with the findings from Peran et al. (2007) and Khodir et al. (2019). Also, Mahoro et al. (2021) found that; DSS-induced bloody diarrhoea, weight loss, shortening of the colon, and mucosal deterioration in a rat model of DSS-induced colitis. Otherwise, only amoxicillin–probiotics treatment could significantly ameliorate the body weight loss and the high stool score caused by DSS/LPS, while both treatments (amoxicillin and probiotics–amoxicillin) showed inhibitory effects on the high colon weight-to-length ratio (*p* < 0.01), as well as colon macroscopic lesion score (*p* < 0.001). In line with these results, Applegate et al. (2010) clarified that; treatment with probiotics resulted in bacterial antagonism, colonization competition, and emulation for nutrients. These actions lead to amelioration of toxic compounds, modulation of the immune system, increasing nutrient absorption and digestibility, and ultimately decline in body weight loss. *Lactobacillus* and *Bifidobacterium* strains, which were used in this study, have anti-inflammatory and immunomodulatory activities. Moreover, certain *Lactobacillus* strains can upregulate the expression of mucin-3 and enhance the intestinal mucus layer (Yao et al 2017), so they could inhibit DSS/LPS-induced damage in the colon tissue.

MPO is a member of the peroxidases subfamily and has more expression in immune cells such as neutrophils, lymphocytes, monocytes, and macrophages. The elevated levels of MPO activity are considered a well-diagnostic marker of inflammation (Khan et al. 2018). In this study, the combined treatment of amoxicillin and probiotics was more effective than amoxicillin alone in ameliorating the elevated MPO activity caused by DSS/LPS. This amelioration is due to the anti-inflammatory properties. The decrease in MPO activity reveals a lower infiltration of neutrophils in the inflamed colon tissue since treatment with probiotics caused a reduction in the colonic production of the chemotactic eicosanoid LTB4. It was observed that; *Lactobacillus acidophilus* administration reduced colonic MPO activity in the trinitrobenzene sulfonic acid (TNBS) model of rat colitis (Peran et al. 2007).

CRP is one of the most prominent proteins in acute inflammation (Norouzinia et al*. *2017). It has been found that higher levels of CRP, as well as the inflammatory markers, have been detected in patients with severe acute diverticulitis disease (Lahat et al. 2019). In the present study, DSS/LPS administration caused a significant elevation (*p* < 0.001) in the serum CRP level. This elevation was declined on either treatment with amoxicillin or probiotics–amoxicillin due to the inhibition of inflammation and disease progression. According to Sartelli et al*.* (2020), CRP is a valuable biomarker of inflammation and can be used in the prediction of the severity of acute diverticulitis. Govindarajah et al. (2014) reported that; there was a significant decrease in serum CRP levels in response to co-amoxicillin gentamycin therapy in a case of appendiceal or acute colonic pathology associated with gall bladder cholecystitis. Similarly, Seaton et al*.* (2020) and Soliman et al*.* (2022) pointed to the role of amoxicillin therapy in decreasing serum CRP levels. Isaacs and Sartor (2004) agreed with these results demonstrating that; antibiotics may decrease bacterial tissue invasion, impair bacterial attachment, decrease bacterial translocation and prevent systemic dissemination. It has been reported that Enterobacteriaceae are essential modulators of colitis. The genus *Klebsiella* promotes the differentiation of T cells and stimulates the production of cytokines, such as (IL)-1β, (IL)-18, (TNF)-α, (IL)-22, and (IL)-17, which promote inflammation. Type 1 helper (T_H_1) and type 17 helper (T_H_17) cells played a pro-inflammatory role in the bacterial-induced intestinal diseases, while regulatory T (Tregs) cells have been shown to have anti-inflammatory and tolerance-maintaining functions (Lin et al. 2020). The current study revealed that DSS/LPS resulted in an elevation of the levels of (TNF)-α, (IL)-1β, (IFN)-γ, and (IL)-18. The pro-inflammatory cytokines such as (IL)-1β and (TNF)-α have been involved in the inflammatory process in DSS-induced colitis (Triantafillidis et al*.* 2011). Lahat et al*.* (2019) demonstrated that; patients with severe acute diverticulitis have higher tissue inflammatory cytokine levels including (TNF)-α, (IL)-6, (IL)-1β, and more inflammatory infiltrates in diverticular colon tissue. On the other hand, the elevation in (TNF)-α, (IL)-1β, (IFN)-γ, and (IL)-18 levels significantly declined after treatment with amoxicillin and probiotics–amoxicillin. Graversen et al. (2020) have found that oral administration of broad-spectrum antibiotics increased the relative abundance of CD4^+^ Treg cells in the small intestine of the Brown Norway rats and reduced the relative abundance of T_H_1 and T_H_17 effector cells. The therapeutic effect of antibiotics in acute diverticulitis disease was explained by Tursi et al*.* (2020) who mentioned that antibiotics led to a decrease in the proportion of T_H_1 cells in the colon. Considering T_H_1 is a pro-inflammatory cell, diverticulitis should be alleviated. This inhibitory effect; in the levels of the pro-inflammatory cytokines can also be attributed to the existence of a cross-talk between probiotics and the mucosal cells. *Bifidobacterium lactis,* either alone or in combination with other probiotics, was able to downregulate the degree of activation of intestinal immune cells in the TNBS model of rat colitis (Peran et al. 2007). Probiotics may restore the balance of gut flora by decreasing pathogenic Gram-negative bacteria that may have been altered in diverticular disease due to stasis and prolonged colonic transit time and have been proposed to be used in diverticular disease to prevent inflammation (Boynton and Floch 2013).

The biochemical analysis and the colonic macroscopic lesions evaluation were confirmed by the histological study, where DSS/LPS caused mucosal and submucosal inflammatory cells infiltration, dilated blood vessels, and degenerative mucosal changes with damaged crypts. Both Schieffer et al. (2018) and Tursi et al. (2020) demonstrated similar changes that occurred in the architecture of the colon wall, including loss of elasticity and deposition of immature collagen fibres in the extracellular matrix, which is implicated in the formation of diverticula as part of the pathophysiology of diverticular disease. Treatment with probiotics–amoxicillin was more effective than treatment with amoxicillin alone in improving the architecture of the colon tissues, since the colon tissues showed slight inflammation and few mucosal lymphocytes infiltration with probiotics–amoxicillin treatment, while showed few mucosal and submucosal inflammatory cells infiltration and degenerative mucosal changes with few damaged crypts with amoxicillin treatment. Conclusively, antibiotics have been used to treat acute diverticulitis in all patients. Recent findings have indicated that the manipulation of antibiotics is not necessary for mild or moderate uncomplicated acute diverticulitis management, as was initially thought (Feuerstein and Falchuk 2016). Treatment of acute diverticulitis generally comprises dietary fibre supplementation, anti-inflammatory drugs, pharmacological therapies such as antibiotics, as well as probiotics, either alone or in combination (Tursi et al*.* 2015). The present study revealed that each of the treatments (amoxicillin and probiotics–amoxicillin) attenuated the severity of DSS/LPS-induced acute diverticulitis in rats and displayed differing effectiveness in disease parameters. Combined probiotics–amoxicillin therapy was more effective in restoring the rats’ body weight, decreasing the high stool score, inhibiting the inflammatory markers tested in this study, and improving the epithelium damage score. Further studies are required to understand how probiotics can be employed in treating acute diverticulitis. The impact of the intestinal milieu, especially enteric microbiota, appears to be of great significance. Our study demonstrates that; probiotics enhanced the prevention of DSS/LPS-induced acute diverticulitis, so we suggest employing probiotics in treating acute diverticulitis disease.

## Data Availability

The data used to support the findings of this study are available from the corresponding author when requested.
